# Supplementary material for: Impact of the COVID-19 pandemic on daily life and worry among mothers in Bhaktapur, Nepal
Source: PLOS Glob Public Health. 2022 Apr 18;2(4):e0000278. doi: 10.1371/journal.pgph.0000278 (PMC10022233; doi:10.1371/journal.pgph.0000278)
Supplement: S1 File — (DOCX) [file pgph.0000278.s001.docx]

**S1 File: Corona Virus Exposure Questionnaire**

| 1 | Where are you living now? 1=Own home, 2= Rent  अहिले तपाइँ कहाँ बस्दै हुनुहुन्छ? १= आफ्नै घर, २= भाँडमा |
| --- | --- |
| 2 | Please check if you have the following members: 1=Yes, 2=No  कृपया जनाउनुहोस् यदि तपाइको परिवारमा निम्न सदस्यहरू छन्: १=छ, २=छैन |
| 2_a | 1= infants ( <1 year ) १ ( शिशुहरू <१ वर्ष |
| 2_b | 2 =Children 1-16 years १ देखि १६ वर्षका बच्चाहरू |
| 2_c | 3 = senior citizen (>60yrs) ५=ज्येष्ठ नागरिक (>६० वर्ष) |
| 3 | Have you faced any of the following symptoms since last two weeks? 1=Yes, 2=No  के तपाईंले गत दुर्इ हप्तादेखि तलका कुनै लक्षणहरूको सामना गर्नुभयो? १=छ, २=छैन |
| 3_a | Fever ज्वरो |
| 3_b | Cold / Cough /Difficulty Breathingखोकी/ रुघा/ श्वास फेर्नमा कठिनाइ |
| 3_c | Sore throat घाँटी दुखेको |
| 3_d | Diarrhea पखाला |
| 4 | Do you have any of the following conditions: 1=present, 2=absent  के तपाईंसँग निम्न अवस्थाहरू मध्ये कुनै छ? १=छ, २=छैन |
| 4_a | Chronic respiratory illness दीर्घकालीन श्वासप्रश्वास रोग |
| 4_b | Renal Disease मृगौला सम्बन्धी रोग |
| 4_c | High Blood Pressure उच्च रक्तचाप |
| 4_d | Diabetes मधुमेह |
| 4_e | Others chronic diseases अन्य दीर्घ रोगहरू |
| 5 | Does your family member have any of the following conditions?1=present, 2=absent  के तपाईको परिवारको सदस्यसँग निम्न अवस्थाहरू मध्ये कुनै छ? १=छ, २=छैन |
| 5_a | Chronic respiratory illness दीर्घकालीन श्वासप्रश्वास रोग |
| 5_b | Renal Disease मृगौला सम्बन्धी रोग |
| 5_c | High Blood Pressure उच्च रक्तचाप |
| 5_d | Diabetes मधुमेह |
| 5_e | Others chronic diseases अन्य दीर्घ रोगहरू |
| 6 | How much were you concerned about the shortage of essential medicines, such as medicine for diabetes, heart diseases, and renal diseases?  1=Not at all, 2=just a little, 3=Some, 4=quite a bit, 5=a great deal, NA=9  मधुमेह, हृदय रोग, र मृगौला सम्बन्धी रोग जस्ता अत्यावश्यक औषधीहरूको अभावको बारेमा तपाईं कत्तिको चिन्तित हुनुभयो?  १= कत्ति पनि छैन, २=अलिकति, ३=केही, ४=धेरै, ५=अत्ति धेरै, ९= लागु नहुने |
| 7 | Were you exposed to someone who was COVID positive within last 2 weeks? 1=Yes, 2=No  के तपाई कसैसँग सम्पर्कमा पर्नु भयो जो कोरोना पोजिटिभ थियो? १=छ, २=छैन |
| 8 | How many days have you been quarantined?  तपाई कति दिन क्वारेनटाइनमा पर्नुभएको छ? |
| 9 | Is there anyone with corona positive in your family? 1=Yes, 2=No  के तपाईको परिवारमा कोरोना पोजिटिभ भएका कोही व्यक्ति हुनुहुन्छ? १=छ, २=छैन |
| 10 | How strongly did you believe that your own life or the life of someone close to you, was threatened or in danger because of Covid-19?  1=Not at all, 2=just a little, 3=Some, 4=quite a bit, 5=a great deal  **COVID-19** को कारणले तपाई वा तपाईको नजिकको व्यक्तिको ज्यान खतरा वा जोखिममा परेको भनेर कत्तिको महशुस गर्नुभयो?  १=कत्ति पनि छैन, २=अलिकति, ३=केही, ४=धेरै, ५=अत्ति धेरै |
| 11 | Has the pandemic a negative effect on your family life?  1=Not at all, 2=just a little, 3=Some, 4=quite a bit, 5=a great deal  के महामारीले तपाईंको पारिवारीक जीवनमा नकारात्मक प्रभाव पारेको छ?  १=कत्ति पनि छैन, २=अलिकति, ३=केही, ४=धेरै, ५=अत्ति धेरै |
| 12 | In which aspect of life has the pandemic affected you? (Enter 9 in Item 12_a to 12_e if the response in 11 is 1).  महामारीबाट तपाईंको जीवनको कुन क्षेत्रमा असर गरेको छ ? (यदि 11 को जवाफ १ छ भने 12_a देखि 12_e मा ९ जनाउनुस्) |
| 12_a | Economy? 1=Not at all, 2=just a little, 3=Some, 4=quite a bit, 5=a great deal  अर्थव्यवस्था ? १= कत्ति पनि छैन, २=अलिकति, ३=केही, ४=धेरै, ५=अत्ति धेरै |
| 12_b | Food security? 1=Not at all, 2=just a little, 3=Some, 4=quite a bit, 5=a great deal  खाद्य सुरक्षा? १= कत्ति पनि छैन, २=अलिकति, ३=केही, ४=धेरै, ५=अत्ति धेरै |
| 12_c | Employment? 1=Not at all, 2=just a little, 3=Some, 4=quite a bit, 5=a great deal  रोजगार? १= कत्ति पनि छैन, २=अलिकति, ३=केही, ४=धेरै, ५=अत्ति धेरै |
| 12_d | Health related issues? 1=Not at all, 2=just a little, 3=Some, 4=quite a bit, 5=a great deal  स्वास्थ्य सम्बन्धि? १= कत्ति पनि छैन, २=अलिकति, ३=केही, ४=धेरै, ५=अत्ति धेरै |
| 12_e | Daily (regular) life? 1=Not at all, 2=just a little, 3=Some, 4=quite a bit, 5=a great deal  दैनिक (नियमित) जीवन? १= कत्ति पनि छैन, २=अलिकति, ३=केही, ४=धेरै, ५=अत्ति धेरै |
| 13 | **Worries related to the pandemic महामारी सम्बन्धि चिन्ता**  Please use the following response option from items 13_a to 13_e:  1=Completely disagree, 2=Disagree, 3=Neither disagree nor agree, 4=Agree, 5=Completely Agree  कृपया 13_a बाट 13_e सम्म निम्न जवाफ प्रयोग गर्नुहोस्:  १= पूर्ण अ सहमत , २= असहमत, ३= न त असहमत न त सहमत, ४= सहमत, ५= पूर्ण सहमत |
| 13_a | I worry that I will contract the corona virus.  मलाई चिन्ता छ कि म कोरोना भाइरस संक्रमित हुनेछु। |
| 13_b | I worry that someone in my family will contract the corona virus.  मलाई चिन्ता छ कि मेरो परिवारमा कसैलार्इ कोरोना भाइरस संक्रमित हुनेछ। |
| 13_c | I sleep worse than before due to worry regarding the health consequences of the Corona virus.  कोरोना भाइरसको स्वास्थ्य परिणामबारे चिन्ताको कारण म पहिले भन्दा झन् खराब निदाउँछु। |
| 13_d | I sleep worse than before due to the social consequences of the Corona virus  कोरोना भाइरसको सामाजिक परिणामहरूका कारण म पहिले भन्दा झन् खराब निदाउँछु। |
| 13_e | I sleep worse than before due to worry regarding the economical consequences of the Corona virus.  कोरोना भाइरसको आर्थिक परिणामहरूको बारेमा चिन्ताले गर्दा म पहिलेको भन्दा खराब निदाउँछु। |
