# Supplementary material for: Impact of the COVID-19 pandemic on daily life and worry among mothers in Bhaktapur, Nepal
Source: PLOS Glob Public Health. 2022 Apr 18;2(4):e0000278. doi: 10.1371/journal.pgph.0000278 (PMC10022233; doi:10.1371/journal.pgph.0000278)
Supplement: S1 Table — (DOCX) [file pgph.0000278.s002.docx]

**S1 Table: Calculation of the WAMI index**

| **Component** | **Description** | **Score range** |
| --- | --- | --- |
| **W**ater and Sanitation | Using World Health Organization definitions of access to improved water and improved sanitation, households with access to safe water or safe sanitation were assigned a score of 3.5 for each. For this context, water from water tanks were also considered as improved. Households without access to improved water or improved sanitation were assigned a score of 0 for each. These scores were summed. | 0-7 |
| **A**ssets | For each assets, households were assigned a 1 if they had the assets and a 0 if they did not have the asset. The following assets were used: used ownership land, ownership house, bedroom/kitchen separate, type cooking fuel, ownership of vehicle, tenants, remittances | 0-7 |
| **M**aternal education | Maternal education was assigned 0 if the mother was illiterate. Formal education was categorized as primary school (3 points), secondary school (4 points), intermediate school (with school leaving certificate) (5 points), bachelor’s degree (6 points), above bachelor’s degree (7 points). | 0-7 |
| **I**ncome | Not available in this study | N/A |
|  |  |  |
| Total WAMI | Scores in water and sanitation, assets, and mother’s education were summed then divided by 21. | 0-1 |
