# Supplementary material for: Impact of the COVID-19 pandemic on daily life and worry among mothers in Bhaktapur, Nepal
Source: PLOS Glob Public Health. 2022 Apr 18;2(4):e0000278. doi: 10.1371/journal.pgph.0000278 (PMC10022233; doi:10.1371/journal.pgph.0000278)
Supplement: S2 Table — (DOCX) [file pgph.0000278.s003.docx]

| **S2 Table: Mean Worry and Sleep scores according to the mode of data collection of Nepalese mothers (n=493)** | | | | | | | |
| --- | --- | --- | --- | --- | --- | --- | --- |
|  |  |  | **Worry Score** | |  | **Sleep Score** | |
| **Variables** | **N** |  | **Mean (SD)** | **p-value** |  | **Mean (SD)** | **p-value** |
| **Mode of data collection** |  |  |  |  |  |  |  |
| In-person | 242 |  | 5.70 (2.64) | 0.005* |  | 4.69 (2.58) | 0.078 |
| By phone call | 251 |  | 5.06 (2.45) |  |  | 5.13 (2.95) |  |
