# Supplementary material for: Impact of the COVID-19 pandemic on daily life and worry among mothers in Bhaktapur, Nepal
Source: PLOS Glob Public Health. 2022 Apr 18;2(4):e0000278. doi: 10.1371/journal.pgph.0000278 (PMC10022233; doi:10.1371/journal.pgph.0000278)
Supplement: S3 Table — (DOCX) [file pgph.0000278.s004.docx]

| **S3 Table: Age and socioeconomic factors according in those that were lost to follow-up compared to those who were included in the study sample.** | | | | | |
| --- | --- | --- | --- | --- | --- |
|  |  |  |  | |  |
| **Variables** | **N** |  | **Mean (SD)** | **p-value** |  |
| **Age of mothers** |  |  |  |  |  |
| Final sample | 493 |  | 32.83 (4.41) | <0.001* |  |
| Lost to follow up | 107 |  | 29.98 (5.04) |  |  |
| **WAMI Score** |  |  |  |  |  |
| Final sample | 493 |  | 0.62 (0.14) | <0.001* |  |
| Lost to follow up | 107 |  | 0.54 (0.14) |  |  |

**WAMI Score:** Measure of household socioeconomic status, including access to improved Water/sanitation, Assets, Maternal education, and Income
