# Supplementary material for: Impact of the COVID-19 pandemic on daily life and worry among mothers in Bhaktapur, Nepal
Source: PLOS Glob Public Health. 2022 Apr 18;2(4):e0000278. doi: 10.1371/journal.pgph.0000278 (PMC10022233; doi:10.1371/journal.pgph.0000278)
Supplement: Attachment — Submitted filename: Response to Reviewers.docx [file pgph.0000278.s005.docx]

**Response letter**

***Thank you very much for the opportunity to revise and resubmit our manuscript and for the thorough review and suggestions. We addressed all the suggestions; below follows a detailed account of our responses and changes to the manuscript.***

Journal Requirements:

1. Please review your reference list to ensure that it is complete and correct. If you have cited papers that have been retracted, please include the rationale for doing so in the manuscript text, or remove these references and replace them with relevant current references. Any changes to the reference list should be mentioned in the rebuttal letter that accompanies your revised manuscript. If you need to cite a retracted article, indicate the article’s retracted status in the References list and also include a citation and full reference for the retraction notice.

*Response: We have added some new references in the updated manuscript and ensured that it is complete and correct.*

2. Please include additional information regarding the survey or questionnaire used in the study and ensure that you have provided sufficient details that others could replicate the analyses. For instance, if you developed a questionnaire as part of this study and it is not under a copyright more restrictive than CC-BY, please include a copy, in both the original language and English, as Supporting Information.

*Response: We have added a full questionnaire in Nepalese and a translated English version as Supplemental table 2.*

3. Please ensure you have included the registration number for the clinical trial referenced in the manuscript.

*Response: We have now included the registration numbers for the clinical trial in the manuscript (Line 102-103)*

4. Please provide separate figure files in .tif or .eps format only, and remove any figures embedded in your manuscript file.

*Response: Now, we have uploaded the figure in .tif format.*

For more information about figure files please see our guidelines: 
<https://journals.plos.org/globalpublichealth/s/figures>

5. Please update the completed 'Competing Interests' statement, including any COIs declared by your co-authors. If you have no competing interests to declare, please state "The authors have declared that no competing interests exist". Otherwise please declare all competing interests beginning with the statement "I have read the journal's policy and the authors of this manuscript have the following competing interests:

*Response: We have updated the statement in the manuscript as suggested (Line 387)*

6. In the online submission form, you indicated that [Insert text from online submission form here]. All PLOS journals now require all data underlying the findings described in their manuscript to be freely available to other researchers, either 1. In a public repository, 2. Within the manuscript itself, or 3. Uploaded as supplementary information.
This policy applies to all data except where public deposition would breach compliance with the protocol approved by your research ethics board. If your data cannot be made publicly available for ethical or legal reasons (e.g., public availability would compromise patient privacy), please explain your reasons by return email and your exemption request will be escalated to the editor for approval. Your exemption request will be handled independently and will not hold up the peer review process, but will need to be resolved should your manuscript be accepted for publication. One of the Editorial team will then be in touch if there are any issues.

*Response: Our data cannot be made publicly available for ethical reasons. We have sent an email explaining the reasons according to your instructions.*

**Data Share Statement:** Data described in the manuscript, code book, and analytic code will be made available upon request pending application and approval by the Nepal Health Research Council (NHRC) and the Regional Committee for Medical and Health Research Ethics in Norway. Requests for data should be sent to the authors, by contacting NHRC (<http://nhrc.gov.np>), or by contacting the Department of Global Health and Primary Care at the University of Bergen ([post@igs.uib.no](mailto:post@igs.uib.no)), or contacting the Department of Pediatrics, Tribhuvan University Institute of Medicine, Kathmandu, Nepal ([chrp2015@gmail.com](mailto:chrp2015@gmail.com)).

.

7. Please amend your] detailed Financial Disclosure statement. This is published with the article, therefore should be completed in full sentences and contain the exact wording you wish to be published.

i) Please include all sources of funding (financial or material support) for your study. List the grants (with grant number) or organizations (with url) that supported your study, including funding received from your institution. 
ii). State the initials, alongside each funding source, of each author to receive each grant.
iii). State what role the funders took in the study. If the funders had no role in your study, please state: “The funders had no role in study design, data collection and analysis, decision to publish, or preparation of the manuscript.”

*Response: We have done our best to fully detail our funding sources according to your instructions in our manuscript (lines 378-385).*

Additional Editor Comments (if provided):

This is an important contribution to our understanding of the COVID-19 pandemic on daily life and well-being. In addition to the suggestions of the reviewers, I would add:

1) Since your framing of the paper and discussion focus on worry, please title the paper accordingly. This is a very interesting framing, and the title should reflect that. Please further incorporate in the introduction that you are considering "worry" not just about COVID-19 itself but many of its sequelae in Nepal (i.e., financial outcomes, loss of childcare, etc.).

Response;

*We have now updated the title and it now reads “Impact of the COVID-19 pandemic on daily life and worry among mothers in Bhaktapur, Nepal”.*

We have also added more information on the sequalae and other aspects in the introduction reading: (Line 54-60)

« The restrictions were related to impacts across many life-domains including economy, health service use and food security. Many Nepalese people lost their jobs as a consequence of the restrictions and lockdowns (6). Mothers of infants were especially hard hit by the imposed restrictions, reflected in the increase in stillbirths and neonatal mortality during the pandemic (7), as well as a reduction in utilization of health services (4), and the losses to production and supply chain of food resulting in food insecurity (8) ».

2) Please indicate the age range of children in the abstract. This is very clear in the methods, but after reading the abstract, I thought certainly outcomes would vary by age of children (which turned out to not be relevant as I saw the study design).

*Response: We have now included the age range of the children in the abstract.*

3) In the discussion, please include more thinking about the gendered experience of women.

*Response: Thank you for the suggestion to include gendered experience of women. We have added some explanation in strengths and limitations (Line 338-341). Read:*

"We have only included mothers in the present study, and whether the results can be generalized to fathers is uncertain. However, since women often have responsibilities for health and care and may also be important role models for children, their perceptions and attitudes may also impact the family’s preventive measures and health.”

Reviewers' comments:

**Comments to the Author**

5. Review Comments to the Author

Please use the space provided to explain your answers to the questions above. You may also include additional comments for the author, including concerns about dual publication, research ethics, or publication ethics. (Please upload your review as an attachment if it exceeds 20,000 characters)

**Reviewer #1:**

This study uses cross-sectional survey data to assess the impact of the COVID-19 pandemic on various aspects of daily life for mothers in Bhaktapur, Nepal. While previous studies in Nepal have assessed psychological impacts during the early pandemic period, this study centers on a later phase of the pandemic (September 2020–February 2021, prior to the predominance of the SARS-CoV-2 Delta variant). Findings of higher pandemic-associated worry in mothers with higher educational attainment and socioeconomic status are concordant with a prior, national-level study assessing pandemic distress in April-May 2020 (Shrestha et al. 2020). Readers of PLOS Global Public Health may be interested in implications of these findings for health education efforts in Nepal, as well as suggestions for addressing these psychological impacts.

Major and minor comments for the authors are provided below.

INTRODUCTION

The authors provide a comprehensive overview of previous literature on the impact of COVID-19 on psychological health in general and in Nepal specifically.

*Response: Thank you very much for the positive feedback.*


Additional contextual information about Bhaktapur municipality (e.g. population demographics, geographical location within Nepal) would be appreciated in the Introduction, as well as in the Discussion of the study findings.

*Response: Now we have updated some contextual information about Bhaktapur municipality in the Introduction (lines 90-93). It now reads:*

*……………..* *”* *The present study was conducted in the Bhaktapur municipality of Nepal. The municipality has a population of 83 000 (1)* *and boarders to the eastern part of Kathmandu, the capital city of Nepal. It is a modern lower income setting characterized by mixed economy in an agriculture-based semi-urban community with many migrant workers from nearby districts”.*

METHODS

The authors have clearly described the participant recruitment strategy and reasons for loss to follow up.

*Response: Thanks for positive feedback.*

Definition of variables is easily understood, but what is meant by “age category of family members” is not immediately clear.

*Response: We have categorized family members into groups according to age based on the vulnerability for serious illness at the initial phase of the pandemic, old age (˃60 year) and children (<16year) were regarded as vulnerable to COVID-19 infection compared to youth and adults.*

*We have updated the manuscript accordingly: « Family members were categorized in age groups for which we considered children <16 and adults >60 more vulnerable to consequences of a COVID-19 infection.» (Line 117-118)*

The methods for statistical analysis are technically sound, although authors should discuss explicitly their strategy for handling any missing data.

*Response: We appreciate the comment on statistical analysis. Regarding missing data, we have included this information in the statistical section. There was very little missing data, and the missing values were handled by listwise deletion.*


The authors received ethical approvals from the Nepal Health and Research Council (NHRC) and Regional Committee for Medical and Health Research Ethics (REC) and described obtaining written consent from study participants. Authors have made data available upon request in accordance with requirements of NHRC and REC.

RESULTS

Results are communicated clearly.

*Response: Thank you.*

Did the authors note any difference in key findings (negative impacts of COVID-19) by mode of data collection (phone vs. in-person interview)?

*Response: The percentage of participants who were interviewed with phone call is now included in the manuscript (Line 140). The analysis showed that the mode of the interview was associated with the worry score but not the sleep score in our study. Those who were interviewed in-person showed higher worry scores compared to those who were interviewed through phone call. It now reads:*

"We also found that the worry score was significantly higher when collecting by an in-person interview compared to through a phone call. The fact that people respond differently if they were interviewed through phone calls rather than through direct contact methods have been demonstrated before "(2) (Please see line 238-240 and line 329-332 and Supplemental Table S3)

TABLES

Tables 1-5 and Supplemental Table 1 are well-organized and easily comprehensible.

*Response: Thanks for positive feedback.*


DISCUSSION/CONCLUSIONS

The authors appropriately situate their findings within a broader discussion of previous literature on pandemic distress in Nepal and in other LMICs.

Authors have thoughtfully considered the Strengths and Limitations of the study, including generalizability, variability in the mode of data collection (phone and in-person interviews), and inability to collect data on depressive symptoms through clinical interview. Authors should additionally address how the study setting in Bhaktapur affects generalizability.

*Response: Thanks for the positive comment. We have expanded the strengths and limitations section and included a section on how our specific study setting may have affected the generalizability of the results (Please see the line 334-341).*

Authors should expound on implications of their findings for readers of PLOS Global Public Health. What are the implications for health education efforts in Nepal if those least concerned about COVID-19 are those who with the lowest educational attainment? What should public health practitioners consider when aiming to address the psychological impacts the findings highlight?

*Response: Thank you very much for the suggestion. Certainly, that those least concerned about COVID-19 are from the group of lower academic qualification might be a challenge when controlling the pandemic. Lower scores on the worry scale may be of concern since this could lead to lower compliance to advice to protect themselves from contracting the virus. At the same time, public health practitioners should consider the level of education while addressing the psychological impacts of pandemics since our results suggests that education can be the deciding factor for psychological impacts of the individuals. We have updated the Discussion part and included some information on this topic (lines 294-299). Read:*

“This group with the lowest educational qualifications being least concerned about contracting COVID-19, might be the challenging factor in controlling the pandemic due to less safety measures to protect them from virus infection. Hence, public health practitioners should consider the level of education while addressing the psychological impacts of pandemics since our results suggests that education can be the deciding factor for any psychological impacts of the individuals.”

FIGURES

Figure 1 is easily comprehensible. The title should be reworded to reflect the participants of the current study, e.g. “Recruitment of mothers in Bhaktapur, Nepal”.

*Response: The suggestion is appreciated and we have changed the title accordingly.*


MINOR COMMENTS:
Use of the term “Corona” should be replaced with “Coronavirus” or “COVID-19,” including in the title of Table 2.

*Response: Thank you very much for suggestion. We have now replaced the term as suggested throughout the manuscript.*


This article satisfies the below criteria for acceptance to PLOS Global Public Health:
1. The study presents the results of primary scientific research.
2. Results reported have not been published elsewhere.
3. Experiments, statistics, and other analyses are performed to a high technical standard and are described in sufficient detail.
4. Conclusions are presented in an appropriate fashion and are supported by the data.
5. The article is presented in an intelligible fashion and is written in standard English.
6. The research meets all applicable standards for the ethics of experimentation and research integrity.
7. The article adheres to appropriate reporting guidelines and community standards for data availability.

**Reviewer #2:**

 The authors present findings of a study that assessed effect of COVID-19 in an urban district of Nepal. The article is largely coherent however it would benefit with some specific detail in few places. I would strongly recommend the article to be professionally edited for language as there are typos and grammatical errors in the article (e.g., line 90, 207, 255-256).

*Response: Thank you for your thorough feedback. We have reviewed the manuscript for language and grammatical errors.*


Line number Comments:
103 Suggest to include some information on characteristics of respondents who were lost to follow up. Was it different from the final sample?

*Response: Thank you very much for your query.*

*We have now compared the responders with the non-responders on key demographic characteristics. Age of the mothers who were lost to follow up was significantly lower than the final sample (p<0.001). In terms of WAMI score, the final sample was better off than those who were lost to follow up (p<0.001).*

*We have updated the manuscript (Line 347-350). Now it reads:*

”The final sample was better off than those who were lost to follow up. The result can be the strength of our study to differentiate the SES indicating that the losses to follow up were mainly due to the difficult financial situation during the lockdown”.

106 Need to specific and consistent in using the term. Caregivers or mothers. Grandparents, father or other members of an extended family can also be a caregiver

*Response: We have only collected data from mothers, and we have now used the term “mothers” throughout the manuscript (line 114).*

113 What was the definition for” no work” specially for mothers (57.8%)? Did it also include housewife or household work? Strongly suggest to use alternative word

*Response: Yes, this category includes housewife and household work that did not get formal payment as the other engaged mothers. Now, we have changed the category as “no formal work”.*

135 Recommend including information tool development of Corona Exposure questionnaire. Was this questionnaire pre-tested?

*Response: The items of Corona Exposure questionnaire had already been used in our previous study in 2015 that was intended to measure the level of exposure to the earthquakes (3) and we used the same for the exposure of Corona Virus. We have updated the manuscript (Line 155-157) and it reads as….*

*“Prior to the study, our study team had thorough discussion of the items in which experienced physicians and psychologists from Nepal and clinical psychologists from Norway were involved.”*

213-215 Convoluted sentence. Please make this sentence easier to read

*Response: We have updated the sentence and it reads as”* *Among the mothers, 37.1% reported worry related to contracting the corona virus and 39.5% reported worry that someone in their family would contract the virus.”. (Line 228-229)*


269 What could be the possible explanation about the low prevalence of sleep problem – What do authors think about that? - more resilience because of repeated stressors? or taking COVID-19 lightly?

*Response: These findings are not easy to explain. It could be that the low prevalence of sleep problem was related to the fact that most of those infected with SARS-CoV-2 during the study period were asymptomatic or had mild symptoms.*

*We have updated the manuscript (Line 284-286)*

287-289 Argument here is really not clear. Please add more reasoning about why they could be less sensitive

*Response: Thanks for feedback. We have updated the manuscript with the brief explanation for being less sensitive and it reads as*

*“……* *due to the lack of health literacy resulting in these being less likely to seek help when they experienced symptoms. Secondly, they might lack the capability of understanding the symptoms and health consequences of the corona virus limiting their interpretation and decision about own health condition (4) .”(Line 309-312)*

**References**

1.	Nepal C. National Population and Housing Census, National Report. Government of Nepal Kathmandu. 2011.

2.	Block ES, Erskine L. Interviewing by telephone: Specific considerations, opportunities, and challenges. International journal of qualitative methods. 2012;11(4):428-45.

3.	Kvestad I, Ranjitkar S, Ulak M, Chandyo RK, Shrestha M, Shrestha L, et al. Earthquake exposure and post-traumatic stress among Nepalese mothers after the 2015 earthquakes. Frontiers in psychology. 2019;10:734.

4.	Zhong B-L, Luo W, Li H-M, Zhang Q-Q, Liu X-G, Li W-T, et al. Knowledge, attitudes, and practices towards COVID-19 among Chinese residents during the rapid rise period of the COVID-19 outbreak: a quick online cross-sectional survey. International journal of biological sciences. 2020;16(10):1745.
